# Importance of the C_12_ Carbon Chain in the Biological Activity of Rhamnolipids Conferring Protection in Wheat against *Zymoseptoria tritici*

**DOI:** 10.3390/molecules26010040

**Published:** 2020-12-23

**Authors:** Rémi Platel, Ludovic Chaveriat, Sarah Le Guenic, Rutger Pipeleers, Maryline Magnin-Robert, Béatrice Randoux, Pauline Trapet, Vincent Lequart, Nicolas Joly, Patrice Halama, Patrick Martin, Monica Höfte, Philippe Reignault, Ali Siah

**Affiliations:** 1 Joint Research Unit N° 1158 BioEcoAgro, Junia, University Lille, INRAE, University Liège, UPJV, University Artois, ULCO, 48, Boulevard Vauban, BP 41290, F-59014 Lille CEDEX, France; remi.platel@junia.com (R.P.); pauline.trapet@junia.com (P.T.); patrice.halama@junia.com (P.H.); 2 ULR 7519—Unité Transformations & Agroressources, University Artois, UniLasalle, F-62408 Béthune, France; ludovic.chaveriat@univ-artois.fr (L.C.); Sarah.LeGuenic@sattnord.fr (S.L.G.); vincent.lequart@univ-artois.fr (V.L.); nicolas.joly@univ-artois.fr (N.J.); patrick.martin@univ-artois.fr (P.M.); 3 Lab. Phytopathology, Department Plants & Crops, Ghent University, B-9000 Ghent, Belgium; Rutger.Pipeleers@UGent.be (R.P.); Monica.Hofte@UGent.be (M.H.); 4 Unité de Chimie Environnementale et Interactions sur le Vivant (EA 4492), Université du Littoral Côte d’Opale, CS 80699, F-62228 Calais CEDEX, France; maryline.magnin-robert@univ-littoral.fr (M.M.-R.); beatrice.randoux@univ-littoral.fr (B.R.); philippe.reignault@univ-littoral.fr (P.R.)

**Keywords:** wheat, *Zymoseptoria tritici*, rhamnolipids, elicitors, structure-activity relationship

## Abstract

The hemibiotrophic fungus *Zymoseptoria tritici*, responsible for Septoria tritici blotch, is currently the most devastating foliar disease on wheat crops worldwide. Here, we explored, for the first time, the ability of rhamnolipids (RLs) to control this pathogen, using a total of 19 RLs, including a natural RL mixture produced by *Pseudomonas aeruginosa* and 18 bioinspired RLs synthesized using green chemistry, as well as two related compounds (lauric acid and dodecanol). These compounds were assessed for in vitro antifungal effect, *in planta* defence elicitation (peroxidase and catalase enzyme activities), and protection efficacy on the wheat-*Z. tritici* pathosystem. Interestingly, a structure-activity relationship analysis revealed that synthetic RLs with a 12 carbon fatty acid tail were the most effective for all examined biological activities. This highlights the importance of the C12 chain in the bioactivity of RLs, likely by acting on the plasma membranes of both wheat and *Z. tritici* cells. The efficacy of the most active compound Rh-Est-C12 was 20-fold lower *in planta* than in vitro; an optimization of the formulation is thus required to increase its effectiveness. No *Z. tritici* strain-dependent activity was scored for Rh-Est-C12 that exhibited similar antifungal activity levels towards strains differing in their resistance patterns to demethylation inhibitor fungicides, including multi-drug resistance strains. This study reports new insights into the use of bio-inspired RLs to control *Z. tritici*.

## 1. Introduction

Wheat is one of the most produced and consumed crops worldwide along with maize and rice. For the 2018–2019 period, the Food and Agriculture Organisation estimated that global wheat production reached 732.1 million tons [1]. This crop is cultivated in many different geoclimatic areas and ecosystems, and it is vastly used for human food as well as livestock feed. Hence, ensuring a safe and sufficient production of this cereal is a vital matter. Although wheat is susceptible to various pathogenic agents, Septoria tritici blotch (STB), caused by the hemibiotrophic fungus *Zymoseptoria tritici*, is considered as the most impacting foliar disease on wheat and is of major concern for wheat production worldwide. Currently, strategies to control this phytopathogen rely mainly on the application of conventional fungicides and, to a lower extent, on the use of partially resistant cultivars. In Western Europe, where the climate is particularly conducive for fungal development, STB is of critical importance, both agronomically and economically, leading to yield losses up to 50%. Thus, 70% of fungicides applied in the European Union are used for the control of this disease, for a cost of approximately 1.2 billion dollars per year [2,3].

The main conventional fungicides used currently for STB control are (i) succinate dehydrogenase inhibitors (SDHIs), carboxamide molecules inhibiting succinate dehydrogenase of the fungal mitochondrial respiratory chain, and (ii) demethylation inhibitor (DMI) fungicides, which target the 14 α-demethylase CYP51 protein involved in the biosynthesis of ergosterol, a major lipid compound of fungal plasma membrane. Although the decreasing sensitivity in *Z. tritici* populations to SDHIs in the field is still being contained, reports regarding the resistance of this fungus towards DMIs have increased in the recent years. Indeed, serious breakdowns in azole fungicide efficacy appeared, firstly in Europe and then all over the world [4,5,6]. Concerning DMI resistance, three mechanisms of resistance have been described: (i) alteration of the CYP51 protein due to mutations in the corresponding gene, causing a decrease of affinity between the fungicide and its target, the protein CYP51, (ii) overexpression of the *CYP51* gene, and (iii) enhanced efflux activity in fungal cells due to the overexpression of genes coding for membrane transporters, leading to populations exhibiting multi-drug resistance (MDR) phenotypes [4]. Because of this fungicide resistance issue and the increasing societal demand to implement a more sustainable agriculture, there is a strong need to develop alternatives to control STB in the field.

In the last decades, many studies have highlighted the potential of green surfactants in agriculture. Biosurfactants are surface-active compounds produced by a wide range of microorganisms. Even though they may be chemically very diverse, they are all amphiphilic molecules, consisting of hydrophobic and hydrophilic moieties. They may be used in agriculture for different applications like bioremediation, stimulating interactions between plants and beneficial microorganisms, as well as in crop protection by acting as biofungicides or/and as plant resistance inducers [7,8,9]. Rhamnolipids (RLs) are secondary metabolites with biosurfactant properties, naturally produced by bacteria belonging to the genera *Pseudomonas* sp. and *Burkholderia* sp. They are naturally biosynthetized as a mixture of compounds with one or two rhamnose residues (mono- or di-RLs), forming a polar hydrophilic head and linked through a beta-glycosylic bond to one or two 3-hydroxy fatty acids, as hydrophobic tails. Rhamnolipids are one of the most intensively studied classes of surfactant glycolipids. Like other biosurfactants, they may potentially be used in a broad range of areas, including medicine, cosmetics, food processing, petroleum industries, and agriculture [10,11,12]. In crop protection, previous studies reported the antimicrobial properties of natural RLs, as well as their capacity to induce plant resistance mechanisms, as reviewed by Vatsa et al. [13]. For instance, they were demonstrated to inhibit the growth of various phytopathogenic fungi, such as *Botrytis cinerea* and *Fusarium solani,* and to control oomycete phytopathogens, such as *Phytophtora* sp., by lysing zoospores [14,15,16,17]. Natural RLs are also able to protect plants, such as grapevine and *Brassica napus*, against *B. cinerea*, by triggering defense responses, such as ROS production [17,18]. Natural RLs were also reported to induce defense mechanisms in *Arabidospis thaliana* against three phytopathogens, including *Pseudomonas syringae* pv. *tomato*, *Hyaloperonospora arabidopsidis*, and *B. cinerea* [19]. RLs are considered as easily biodegradable and exhibit low toxicity and ecotoxicity, making them promising compounds for plant protection [20,21].

Nevertheless, even though RLs are known to be synthesized by various bacterial species, they are still mostly produced using *Pseudomonas aeruginosa*, a species that cannot be used as biocontrol agent because it is an opportunistic human pathogen. In addition, large-scale industrial production is difficult because of low yields obtained with *P. aeruginosa*. To overcome these issues, three methods may be proposed, such as (i) the use of non-pathogenic natural RLs-producing bacteria such as *Burkholderia* sp. [22], (ii) their heterologous synthesis with genetically modified microorganisms generally recognized as safe, such as *Saccharomyces cerevisiae* [23], or (iii) their synthesis using green chemistry. Recently, synthetic environmentally-friendly RLs produced by green chemistry were found to be able to trigger ROS production in *A. thaliana,* showing a promising potential as plant resistance inducers [24]. Moreover, Robineau et al. [25] demonstrated that synthetic mono-RLs confer protection to tomato against *B. cinerea* by both direct antifungal activity and host defense mechanism activation.

So far, biological activity of RLs has never been explored in the wheat-*Z. tritici* pathosystem. The objective of the present study was thus to assess the direct antifungal activity, the plant eliciting effect, as well as the protection efficacy of 19 RLs (either natural, produced by *P. aeruginosa*, or synthetic (bio-inspired) obtained by green chemistry), as well as lauric acid and dodecanol (C_12_-carbon chain molecules) on the wheat-*Z. tritici* model. A structure-activity relationship analysis of these compounds was performed in order to highlight the chemical radicals responsible for the RL activities. Moreover, the efficacy of the most active RL was compared between in vitro (direct antifungal activity) and *in planta* (disease symptom reduction) conditions. Finally, a possible strain-dependant effect of the RL activity was investigated by examining the in vitro antifungal activity of the most active RL toward 21 *Z. tritici* strains that differ in their resistance level to DMI fungicides.

## 2. Results

### 2.1. Chemical Description of the Synthesized RLs

A total of 18 RLs were obtained using green chemistry to investigate their biological activities on the wheat-*Z. tritici* pathosystem (Table 1). Three series of rhamnose derivatives, differing by the function linking the hydrophobic chain (ether, ester, or succinates), were successfully synthesized. A first series of ether rhamnose derivatives (Rh-Eth), synthesized without using solvent from the _L_-rhamnose and a fatty alcohol with various chain lengths (four to 18 carbon atoms), was produced (Table 1). Then, a second series of RLs, with an ester link (Rh-Est), was obtained (eight to 12 carbons). Finally, succinate rhamnose derivatives (Rh-Suc), including monorhamnosyl (alkenyl)succinate (Rh-Suc-C8, Rh-Suc-C12, and Rh-Suc-C12b) and monorhamnosyl (alkenyl)alkylsuccinate (Rh-Suc-C8-C8, Rh-Suc-C8-C12, Rh-Suc-C12-C8, and Rh-Suc-C12-C12) derivatives, were generated (Table 1). Among the synthesized RLs, nine of them (Rh-Eth-C4, Rh-Eth-C6, Rh-Eth-C8, Rh-Eth-C10, Rh-Eth-C12, Rh-Eth-C14, Rh-Eth-C16, Rh-Est-C12, and Rh-Suc-C12) have already been reported [25], while the nine other RLs, belonging for a large part to the new RL family mono-rhamnosyl (alkenyl)alkylsuccinate, were obtained for the first time (Rh-Eth-C18, Rh-Est-C8, Rh-Est-C10, Rh-Suc-C8, Rh-Suc-C12b, Rh-Suc-C8-C8, Rh-Suc-C8-C12, Rh-Suc-C12-C8, Rh-Suc-C12-C12). For monorhamnosyl (alkenyl)-alkylsuccinate RLs, they were obtained after esterification of _L_-rhamnose with octenylsuccinic or dodecenylsuccinic anhydrid in *N,N-*dimethylformamide (DMF) in presence of *N,N*-dimethylpyridin-4-amine (DMAP), followed by a second esterification by using alkyl bromide. Then, by combining the use of an alkylated succinic anhydride with 8 or 12 carbon atoms and an octanoic or lauric acid chloride during the synthesis from rhamnose, four new derivatives of rhamnose (Rh-Suc-C8-C8, Rh-Suc-C8-C12, Rh-Suc-C12-C8 and Rh-Suc-C12-C12)**,** with a yield comprised between of 34 and 39%, were successfully developped.

### 2.2. Biological Activities of RLs Differ According to the Length of Their Carbon Chain

**Figure 1:**
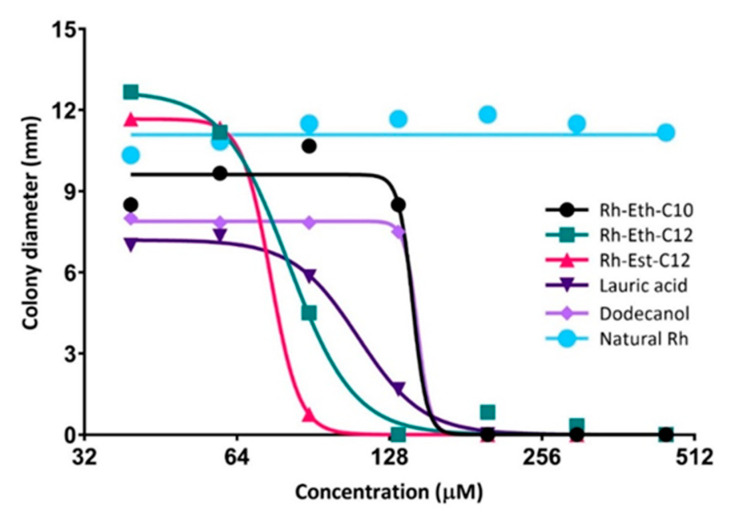
In vitro dose-response curves displaying the antifungal effect of the three most efficient RLs, as well as dodecanol and lauric acid, against *Zymoseptoria tritici* (strain T02596) compared to the natural RLs.

A total of 21 molecules, including 18 synthesized RLs and three related molecules (a mixture of natural RLs, lauric acid, and dodecanol) were examined for both their direct (antifungal) and indirect (plant defence elicitation) activities, as well as for their protection efficacy in the greenhouse, on the wheat-*Z. tritici* pathosystem. Regarding the in vitro antifungal activity, among all tested RLs, from C4 to C18-derived molecules, only Rh-Eth-C10, Rh-Eth-C12, and Rh-Est-C12 drastically inhibited fungal growth (Table 2, Figure 1). Succinate-RLs did not significantly reduce fungal growth, except for Rh-Suc-C8, which showed an inhibitory effect only at the highest tested concentration of 1500 μM (Table 2). Concerning the ester-RL family, Rh-Est-C10 had no antifungal activity, while Rh-Est-C12 was found to be the most active molecule among all tested RLs (Figure 1). On the other hand, lauric acid and dodecanol displayed a marked in vitro antifungal effect againt *Z. tritici*, with IC_50_ and MIC values close to those of the active RLs. Natural RLs did not show any direct antimicrobial effect.

Concerning the ability of the molecules to elicit plant defenses, activity of two plant enzymes involved in ROS scavenging, catalase (CAT) and peroxidase (POX), was investigated two days after treatment. Only three molecules significantly modified at least one enzymatic activity (Table 2). Rh-Eth-C12 increased CAT activity by 1.86 fold compared to the control, whereas Rh-Suc-C12b enhanced POX activity by 2.26 fold compared to the control. Rh-Suc-C12, whose chemical structure is close to Rh-Suc-C12b, did not exhibit any induction of the targeted enzymes in our conditions. Finally, Rh-Est-C12 induced both CAT activity and POX activities by 1.71 and 2.27 folds, respectively, when compared to the control. Lauric acid and dodecanol did not cause any significant change in the investigated enzyme activities.

None of the tested molecule displayed *in planta* phytotoxicity at the tested concentration (1.5 mM). Five of the tested RLs caused a significant reduction in disease symptoms (Table 2). Among them, the four molecules Rh-Eth-C12, Rh-Suc-C12b, Rh-Eth-C10, and Natural-Rh conferred only a slight protection effect, with a decrease in disease severity ranging from 15.5 to 17.4%. On the other hand, disease severity reduction obtained with Rh-Est-C12 treatment was significantly different from all other treatments and exhibited the highest activity, with 35.6% of disease symptom reduction (approximatively 2 fold higher than the protection efficacies conferred by the other active RLs). On the contrary to RLs with C12-carbon chain, lauric acid and dodecanol did not display any significant protection activity.

### 2.3. Correlations between In Vitro Antifungal Activity, in Planta Defence Elicitation and Protection Efficacy

A correlative analysis was performed to determine whether in vitro antifungal and elicitation activities were relevant to explain disease symptom reduction caused by the treatments. The statistical analyses revealed significant positive correlations between both IC_50_ and MIC and the disease severity reduction (r = 0.46) (Table 3). However, no significant correlation was found between CAT activity and protection efficacy, while POX activity and disease symptoms were significantly and negatively correlated (r = −0.51).

### 2.4. Comparison of In Vitro Versus in Planta Antifungal Effects of Rh-Est-C12

**Figure 2:**
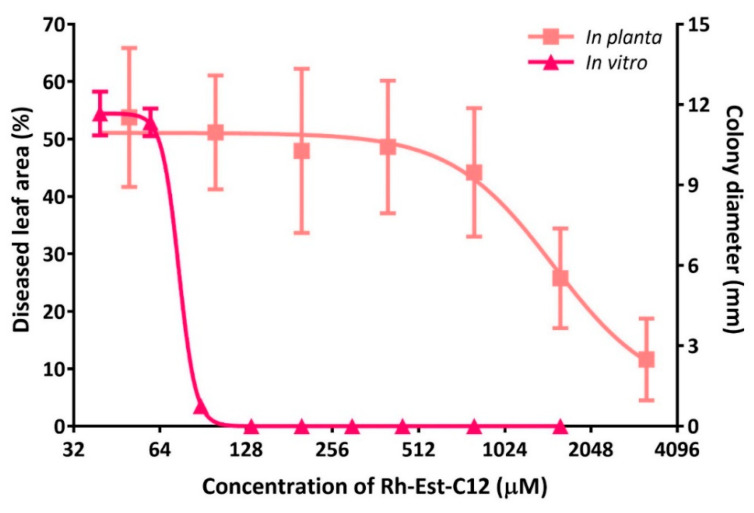
Comparison of *in planta* and in vitro dose-response curves of Rh-Est-C12 on the wheat-*Zymoseptoria tritici* pathosystem. Wheat cultivar Alixan and *Z. tritici* strain T02596 were used. The *in planta* activity of the compound was assessed by measuring the percentage of chlorosis and necrosis on the third leaves of each plant at 21 days after inoculation. Bars stand for standard deviations.

According to the structure-activity relationship study above, Rh-Est-C12 appeared to be the most active RL for protection efficacy, in vitro antifungal effect, and elicitation activity. Hence, we focused on this molecule in order to compare its direct activity in vitro and in planta. In planta, the protective effect of foliar treatment with Rh-Est-C12 on wheat against *Z. tritici* was assessed at different concentrations. There was no significant protection with applications of Rh-Est-C12 at 50, 100, 200, and 400 µM of the RL. However, Rh-Est-C12 treatments with 800, 1600, and 3200 µM significantly reduced disease symptoms on wheat leaves, with protection rates of 19.9%, 53.3%, and 78.9% respectively (Figure 2). No phytotoxicity was observed at any of the tested concentrations. The *in planta* IC_50_ value of this molecule (the concentration of Rh-Est-C12 reducing by half the disease symptom level compared to the non-treated control) was calculated and was determined as 1510 µM. Rh-Est-C12 exhibited an *in planta* IC_50_ value approximatively 20-fold higher than the IC_50_ value obtained in vitro for the molecule, revealing a major difference in the activity of the molecule between the two conditions.

**Figure 3:**
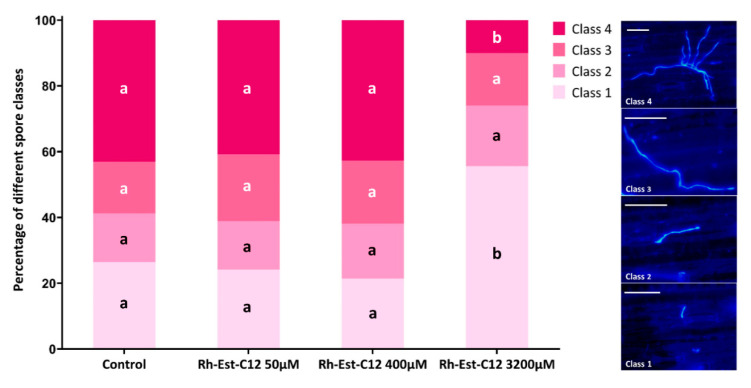
*In planta* spore germination and hyphal growth of *Zymoseptoria tritici* on wheat leaves treated or not with Rh-Est-C12. Wheat cultivar Alixan and *Z. tritici* strain T02596 were used. Percentage of four different classes of Calcofluor-strained spores was assessed three days after inoculation on wheat third leaves. The right panel shows a representative picture of each spore class (scale bar = 25 μm). Class 1: non germinated spore; Class 2: geminated spore with small germ tube; Class 3: geminated spore with developed germ tube; Class 4: geminated spore with a strongly developed germ tube. For each condition, 100 different spores from six different leaves were assessed. Within each class, different letters indicate significant differences between treatments according to ANOVA followed by the Tukey’s test at *p* ≤ 0.05.

Additionally, the effect of Rh-Est-C12 on *in planta* spore germination and fungal hyphal growth was assessed at three different concentrations (50, 400, and 3200 µM) (Figure 3). Whereas no significant activity was observed after application of Rh-Est-C12 at 50 and 400 µM, a strong antifungal effect was highlighted for this compound at 3200 µM. The proportion of non-germinated spores in the treated plants was 2-fold higher than in the control. Moreover, a reduction by 4-fold of the class 4 spore proportion (geminated spores with a strongly developed germ tube) was also scored in the plants treated with 3200 µM Rh-Est-C12 compared to the control (Figure 3). Again, the *in planta* direct activity of Rh-Est-C12 on hyphal growth appeared weaker than its direct antifungal effect obtained in vitro on fungal colony development.

### 2.5. Direct In Vitro Antifungal Activity of Rh-Est-C12 Does not Vary Depending on Z. tritici Strains

**Figure 4:**
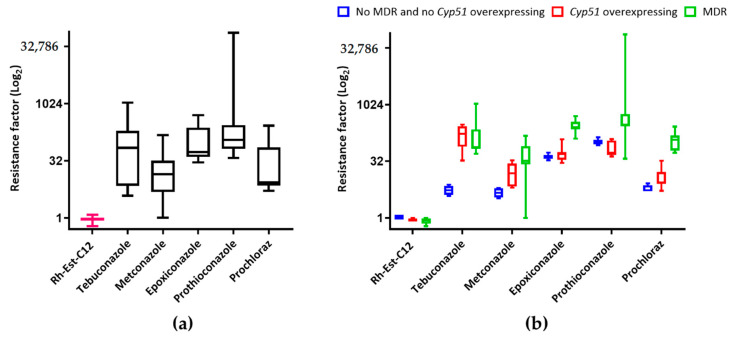
Boxplots showing the resistance factors obtained for 21 different *Zymoseptoria tritici* strains analyzed together (**a**) or distinguished according to their DMI resistance profiles (seven MDR strains, seven strains overexpressing the *Cyp51* gene, and seven strains without these resistance mechanisms) (**b**), against five DMI fungicides as well as the RL molecule Rh-Est-C12. Resistance factors correspond to the ratio between the IC_50_ value of a given strain and the IC_50_ value of the sensitive reference strain IPO323.

To examine any possible strain effect of Rh-Est-C12 direct in vitro antifungal activity, 21 different *Z. tritici* strains showing different resistance levels towards DMI fungicides, were investigated for their resistance level to this RL. Overall, no major difference was observed between all tested strains regarding the IC_50_ values of Rh-Est-C12 (Figure 4A, Tables S1 and S2). Resistance factors (ratio between the IC_50_ value of a given strain and the IC_50_ value of the sensitive reference strain IPO323) ranged from 0.7 to 1.2 for Rh-Est-C12, indicating no strain effect on the direct activity of the molecule. By contrast, a strong strain effect was highlighted for the five tested DMI fungicides, with resistance factors ranging from 3.8 to 1071.4 for tebuconazole, from 1.0 to 150.8 for metconazole, from 28.6 to 501.8 for epoxiconazole, 37.4 to 74753.2 for prothioconazole, and from 5.1 to 266.2 for prochloraz (Figure 4A, Tables S1 and S2). Rh-Est-C12 displayed similar antifungal activity levels on both (i) non-MDR and non *Cyp51-*overexpressing strains, and (ii) the highly resistant strains with MDR character or overexpressing the *Cyp51* gene (Figure 4B, Tables S1 and S2).

## 3. Discussion

We explored here, for the first time, the potential of RLs to protect wheat against *Z. tritici*, a major pathogen on wheat crops, using a panel of 21 molecules, including 18 bioinspired RLs synthesized using green chemistry and three related molecules (a mixture of natural RLs from *P. aeruginosa*, lauric acid, and dodecanol). Natural bacterial RLs have already been reported to show antimicrobial activity against a wide range of plant pathogenic fungi [13]. For instance, Varnier et al. [17] reported a significant antigerminative effect of 0.1 mg·mL^−1^ of bacterial RL against the ascomycete polyphagous fungus *B. cinerea*. Moreover, natural RLs were found particularly effective against certain oomycetes, such as *Phytophthora* sp., by lysing zoospores, hypothetically by intercalating into oomycete cell membranes, leading to their disorganization [14]. However, in our conditions, and even with concentrations up to 0.87 mg·mL^−1^ (i.e., 1500 µM) no activity was observed for the tested mixture of natural RLs. Among all tested compounds, only a minority exhibited significant in vitro antifungal activity. The structure-activity relationship analysis revealed that bioinspired RLs with 4, 6, 8, 14, 16, and 18 carbon fatty acid tails did not display any significant direct effect. Similarly, succinate-derived molecules were also unable to inhibit fungal in vitro growth, except Rh-Suc-C8 which exhibited a very slight effect. The most effective RLs were ether and ester-derived compounds, namely Rh-Eth-C10, Rh-Eth-C12, and Rh-Est-C12. Notably, Robineau et al. [25] also described the antifungal activity of these three compounds against *B. cinerea*. However, they observed a more pronounced effect with Rh-Eth-C10. In the present work, Rh-Eth-C10 was not as effective as Rh-Est-C12 that displayed the lowest IC_50_ among all the tested active compounds (158 and 75 μM, corresponding to 0.048 and 0.026 mg·mL^−1^, for Rh-Eth-C10 and Rh-Est-C12, respectively) (Table 1). Moreover, Rh-Est-C10 did not exhibit any inhibition of fungal growth. Hence, we conclude that 12 carbon is the optimum fatty acid tail length for both ether and ester-RLs regarding their direct activity against *Z. tritici*. Thereafter, we assessed the antifungal potential of chemically simple molecules with 12 carbon chain, lauric acid and dodecanol. Interestingly, these two molecules exhibited a significant direct effect, but their IC_50_ are higher than those of Rh-Est-C12 and Rh-Eth-C12 (Table 2). These results confirm the importance of the 12-carbon chain in the direct activity of RLs towards *Z. tritici*.

We also investigated the elicitor potential of RLs on non-infected wheat using two key enzyme biomarkers of plant defence. Only three molecules significantly enhanced the activity of at least one out of the two targeted plant defense-related enzymes CAT and POX. Rh-Eth-C12 was able to stimulate CAT activity, Rh-Suc-C12b induced POX activity, and Rh-Est-C12 increased both (Table 2). These enzymes are involved in many plant defense responses including ROS scavenging, plant signaling, and hypersensitive response [26,27]. These results suggest an increase of ROS production in wheat plants treated with these three compounds. Since large amounts of ROS in plant tissues can be harmful to the plant, wheat plants likely responded by increasing the activity of ROS scavenging enzymes. Interestingly, natural and synthetic RLs were reported to confer resistance and trigger oxidative burst in other plants such as *A. thaliana* and grapevine [17,24]. Robineau et al. [25] also described a significant increase of ROS production in *A. thaliana* leaf disks treated with Rh-Eth-C12 and Rh-Est-C12. Accumulation of H_2_O_2_ in wheat leaves during infection with *Z. tritici* was reported to increase plant resistance against the pathogen [28]. While the role of CAT in wheat defense against *Z. tritici* is still unclear, Shetty and colleagues [29] reported an involvement of POX in wheat innate immunity. The authors observed a significant induction of this enzyme in resistant cultivars when infected with *Z. tritici*, compared to susceptible cultivars. This may be explained by the specific roles of POX, such as the reinforcement of cell walls and deposition of polyphenolics [30]. This link between POX and innate resistance of wheat to *Z. tritici* is supported by the positive correlation scored between the induction of POX enzymatic activity and plant protection, i.e., negative correlation between POX activity and disease symptoms (Table 3).

As for the in vitro direct antifungal activity, interestingly, our results revealed that RLs with C12 fatty acid chain were the most effective regarding the induction of the targeted wheat defense biomarkers (CAT and POX). This dual activity on both the host (wheat) and the pathogen (*Z. tritici*) is more likely due to RL interaction with biological plasma membranes of both organisms. Indeed, Monnier and colleagues [31] demonstrated, using in silico models, that RLs can act on both plant and fungal membranes by insertion within the lipid bilayers, near the lipid phosphate group of the phospholipid bilayers, nearby phospholipid glycerol backbones. Even though RLs fit into both membranes, RLs seem to differently affect their fluidity. Indeed, the authors suggested that, in the plant membrane, RLs did not significantly impact lipid dynamics inside the lipid bilayer, whereas, in the fungal membrane, a clear fluidity increase was observed, due probably to the presence of ergosterol. These subtle changes in lipid dynamics could explain why the effect of RLs is less drastic on the plant (only triggering defense reactions) than on the fungus that leads to a strong membrane destabilization causing cell death [31]. The importance of the RL carbon chain in plant defense activation has already been reported in previous studies. For instance, Luzuriaga-Loaiza et al. [24] highlighted different results with synthetic RL bolaforms (SRBs) in *A. thaliana*, where SRBs with 14-carbon fatty acid tails were found to be the most effective to induce ROS production in the plant petioles. Regarding other surfactant agents, it has been shown that for surfactin, a lipopeptide produced by *Bacillus subtilis*, the molecules with chain length of 14 or 15 carbons were found more active than surfactin with 12 carbons to enhance production of H_2_O_2_ in tobacco cells [32]. On the other hand, we found that lauric acid and dodecanol were not able to elicit POX- and CAT-based defense reactions, suggesting a major role of the sugar head in plant defense induction. Although further investigations are needed to decipher the modes of action of RLs on wheat defense induction, such a result suggests the major importance of both rhamnose head and fatty acid tail in the biological activities of RLs on this plant model. Moreover, the precise structure of the RL carbon chain is also crucial, since we observed a significant enhancement of POX activity when using Rh-Suc-C12b compared to its isoform Rh-Suc-C12, which showed no elicitation activity in the treated wheat plants.

Our results revealed that Rh-Eth-C10, Rh-Eth-C12, Rh-Est-C12, Rh-Suc-C12b, and natural RLs are able to reduce *Z. tritici* disease severity on wheat plants. Overall, four categories of protective RLs were observed: (i) protective RLs exhibiting only antifungal activity (Rh-Eth-C10); (ii) protective RLs displaying only elicitation effect (Rh-Suc-C12b); (iii) protective RLs with both activities (Rh-Eth-C12 and Rh-Est-C12), and (iv) protective RLs displaying neither direct nor indirect effects (Natural-Rh), implying that this latter compound could induce plant defenses through mechanisms not involving our two targeted enzymatic biomarkers. Statistical analyses revealed a positive correlation between POX activity and protection efficacy, as well as between in vitro direct activity and protection efficacy. Surprisingly, even though lauric acid and dodecanol were able to inhibit fungal growth in vitro with IC_50_ values closes to those of protective RLs, they were unable to protect wheat against the disease, highlighting again the role of rhamnose head in RL protective activity. As discussed above, our structure-activity relationship study reveals the predominant importance of 12 carbon chain in RL biological activities in the wheat-*Z. tritici* model. Likewise, Robineau et al. [25] also described the important role of 12 carbon fatty acid tails in the bioactivity of RLs on the *A. thaliana*–*B. cinerea* pathosytem.

The mono-RL Rh-Est-C12 was found to be the most effective RL to protect wheat against *Z. tritici*, displaying a reduction of disease symptoms up to 78.9% when applied at 1.1 mg·mL^−1^ (i.e., 3200 µM). This product is able to act dually, by inhibiting fungal growth and eliciting plant defenses by enhancing ROS scavenging enzyme activities. Nevertheless, a significant gap between the in vitro and *in planta* efficacies of this molecule was highlighted. A ratio of approximately 20-fold separates the in vitro IC_50_ value of Rh-Est-C12 and the needed concentration to reduce disease symptoms by 50% *in planta*. This difference of activity may be explained by several factors, including the adherence level of Rh-Est-C12 on wheat leaves during spraying, biodegradability as well as photodecomposition, and penetration properties of the molecule in wheat leaves. Further studies on formulation may increase and optimize the *in planta* efficacy of Rh-Est-C12. Another explanation of the gap between in vitro and *in planta* activities of Rh-Est-C12 is the difference between the physiological state of the fungus in vitro and *in planta*. Indeed, the fungus develops *in planta* hyphae which can be considered as more robust (since it is in its wild environment, i.e., within its host) than the yeast-like morphotype formed by *Z. tritici* in vitro. Another major difference between in vitro and *in planta* conditions is that *Z. tritici* develops *in planta* several specialized structures such as pseudo-appressoria and pycnidia that it cannot form in vitro, because of the absence of the host in this latter condition.

Considering the high diversity of *Z. tritici* populations that can be found in a single geographic area [33], we assessed the variability of the direct antifungal activity of Rh-Est-C12 towards 21 different strains of the fungus differing in their resistance level to DMI fungicides, the most used chemical class in the field to control this pathogen. Although the in vitro IC_50_ value of Rh-Est-C12 was, as expected, much higher than those of conventional fungicides on the reference strain IPO323 (54.3 µg·mL^−1^, i.e., 157 µM, for the RL vs. 0.001 to 0.07 μg·mL^−1^ depending on the considered fungicide, Table S1), it is notherworthy that no strain effect was observed for Rh-Est-C12. By contrast, strong resistance factors were scored for all tested DMI molecules, thus confirming previous studies reporting high rates of DMI resistance in *Z. tritici* populations [34]. Hence, we could assume that Rh-Est-C12 display a mode of action on *Z. tritici* different from that of DMI fungicides, which inhibit the sterol 14 α-demethylase, CYP51, preventing the fungus to produce ergosterol [35]. Here, we suggest that Rh-Est-C12 interacts directly with *Z. tritici* cell membranes, resulting in an inhibition of the fungal growth, as previously suggested for other fungi [14,18].

The environmentally-friendly properties of RLs, the duality of Rh-Est-C12 biological activities (direct and indirect activities), an absence of strain specific resistance within the examined *Z. tritici* collection, together with the low-cost production and industrial feasibility of bioinspired synthetic RLs compared to bacterial production and purification, makes Rh-Est-C12 a promising candidate to control *Z. tritici* in the field. Nevertheless, additional investigations on the formulation aiming at reducing the concentration of the compound *in planta* are required.

## 4. Materials and Methods

### 4.1. Chemical Compounds Used

A total of 18 RLs varying in their structures (chain length and/or linker) synthetized using green chemistry, as well as lauric acid and dodecanol (Sigma-Aldrich, Saint Quentin Fallavier, France), were used in order to investigate the structure-activity relationship of RLs on the wheat-*Z. tritici* pathosystem (Table 1). Moreover, commercial RLs purchased from Agae Technologies LLC (Corvallis, OR, USA) were used as a natural RL reference. This natural RL is a mixture of RLs of different structures, composed by decanoic acid, 3-((6-deoxy-2-O-(6-deoxy-α-_L_-mannopyranosyl)-α-_L_-mannopyranosyl)oxy)-, 1-(carboxymethyl)octyl ester (Rh-Rh-C10-C10), and 1-(carboxymethyl)octyl 3-((6-deoxy-α-_L_-manno- pyranosyl)oxy)decanoate (Rh-C10-C10), and produced by *P. aeruginosa* with a purification rate of 90% (data from Agae Technologies LLC). According to the provided information, its average molar mass is estimated to 577.72 g·mol^−1^.

The conversions and the purities of the synthetized products were determined by NMR spectroscopy. NMR spectra were recorded on a DRX300 spectrometer (Bruker, Wissembourg, France) operating at 300 MHz for ^1^H nuclei and 75 MHz for ^13^C nuclei. CDCl_3_ (99.50% isotopic purity) were purchased from Euriso-Top (Saint Aubin, France). The progress of the reactions was checked by thin layer chromatography (TLC) on silica gel 60 glass plates (Merck, Fontenay-sous-Bois, France). Detection was carried out by spraying the chromatograms with 10% ethanolic sulfuric acid and heating them to 100 °C. Flash column chromatography was performed with silica gel (40–100 μm, Merck): all chemicals were of reagent grade and used without further purification.

### 4.2. General Procedures for RL Synthesis

Among the 18 synthesized RLs, nine belonging to ether (butyl α/β-_L_-rhamnopyranoside (Rh-Eth-C4), hexyl α/β-_L_-rhamnopyranoside (Rh-Eth-C6), octyl α/β-_L_-rhamnopyranoside (Rh-Eth-C8), decyl α/β-_L_-rhamnopyranoside (Rh-Eth-C10), dodecyl α/β-_L_-rhamnopyranoside (Rh-Eth-C12), tetradecyl α/β-_L_-rhamnopyranoside (Rh-Eth-C14), hexadecyl α/β-_L_-rhamnopyranoside (Rh-Eth-C16)), ester (dodecanoyl α/β-_L_-rhamnopyranoside (Rh-Est-C12)), and monorhamnosyl (alkenyl)succinate (dodecenylsuccinate α/β-_L_-rhamnopyranoside (Rh-Suc-C12)) families, have already been described in Robineau et al. [25]. The nine new synthesized RLs in the current study were octadecyl α/β-_L_-rhamnopyranoside (Rh-Eth-C18) (ether family), octanoyl α/β-_L_-rhamno-pyranoside (Rh-Est-C8), decanoyl α/β-_L_-rhamnopyranoside (Rh-Est-C10) (ester family), octenylsuccinate α/β-_L_-rhamnopyranoside (Rh-Suc-C8), iso-dodecenylsuccinate α/β-_L_-rhamnopyranoside (Rh-Suc-C12b) (monorhamnosyl (alkenyl)succinate family), 3-octenyloctylsuccinate α/β-_L_-rhamnopyranoside (Rh-Suc-C8-C8), 3-octenyldodecyl-succinate α/β-_L_-rhamnopyranoside (Rh-Suc-C8-C12), 3-dodecenyloctylsuccinate α/β-_L_-rhamnopyranoside (Rh-Suc-C12-C8), and 3-dodecenyldodecylsuccinate α/β-_L_-rhamnopyranoside (Rh-Suc-C12-C12) (monorhamnosyl (alkenyl)alkylsuccinate family).

The general procedures for rhamnose ether, ester and monorhamnosyl (alkenyl)succinate molecule syntesesis have been reported in Robineau et al. [25], while the general procedure for monorhamnosyl (alkenyl)alkylsuccinate derivative synthesis will be described as follow. First, alkenylsuccinic anhydride (12.2 mmol, 1 eq.) was added dropwise to a stirred solution of _L_-rhamnose (2.0 g, 12.2 mmol, 1 eq.) and DMAP (1.8 g, 14.6 mmol, 1.2 eq.) in DMF (20 mL). After 16 h at 60 °C, DMAP (1.5 g, 12.2 mmol, 1 eq.) and alkyl bromide (14.6 mmol, 1.2 eq.) was added slowly to the reaction mixture and stirred overnight. The reaction was cooled and H_2_SO_4_ 10% was added to reached pH 5. Ethyl acetate was added and the organic phase was washed with a saturated NaCl solution and HCl 1 M. The organic layer was dried with MgSO_4_ and concentrated under reduced pressure. The rhamnose alkylsuccinate was isolated after purification by column chromatography (EtOAc/Petroleum spirit 5:5).

### 4.3. RL Chemical Analysis

The chemical analyses of the previously reported RLs Rh-Eth-C4, Rh-Eth-C6, Rh-Eth-C8, Rh-Eth-C10, Rh-Eth-C12, Rh-Eth-C14, Rh-Eth-C16, Rh-Est-C12, Rh-Suc-C12 were already performed [25]. For the nine new synthesized RLs in the present study, their chemical analyses are described below. Confirmatory analyses included elemental analysis (vario MICRO cube CHNS/O, Elementar, Lyon, France); and nuclear magnetic resonance spectroscopy (NMR, Bruker 400 MHz spectrometer).

#### 4.3.1. Octadecyl α/β-_L_-Rhamnopyranoside (Rh-Eth-C18)

White solid (β/α ratio 2:8, 30%); Rf 0.70 (EtOAc/MeOH 9/1);^1^H-NMR (CDCl_3_): 4.73 (m, 2H), 3.91 (m, 2H), 3.77–3.73 (m, 2H), 3.66–3.59 (m, 4H), 3.46–3.34 (m, 4H), 1.57–1.53 (m, 4H), 1.31–1.25 (m, 66H), 0.87 (m, 6H, CH_3_) ^13^C-NMR (CDCl_3_): δ 100.3, 99.6, 73.4, 72.4, 71.4, 70.9, 69.7, 68.2, 68.1, 31.0–21.9 (CH_2_ alkyl chain), 17.9, 17.3, 14.4. Elemental Analysis; calculated for C_24_H_48_O_5_: C, 69.19; H, 11.61; O, 19.20; found: C, 69.27; H, 11.60; O, 19.13.

#### 4.3.2. Octanoyl α/β-_L_-Rhamnopyranoside (Rh-Est-C8)

Yellowish oil (β/α ratio 3:7, 61%); Rf 0.41 (EtOAc/MeOH 9/1); ^1^H-NMR (CDCl_3_): 5.06–4.79 (m, 2H), 4.06–3.87 (m, 4H), 3.63–3.41 (m, 4H), 2.41–2.35 (m, 4H), 1.63–1.59 (m, 4H), 1.31–1.23 (m, 22H), 0.92 (m, 6H, CH_3_) ^13^C-NMR (CDCl_3_): δ 174.2, 172.0, 93.2, 92.9, 75.8, 74.2, 73.3, 72.8, 71.1, 69.8, 68.6, 68.1, 34.5, 34,1, 32.0–21.6 (CH_2_ alkyl chain), 17.5, 17.1, 13.3. Elemental Analysis; calculated for C_14_H_26_O_6_: C, 57.91; H, 9.03; O, 33.06; found: C, 57.85; H, 9.11; O, 33.04.

#### 4.3.3. Decanoyl α/β-_L_-Rhamnopyranoside (Rh-Est-C10)

Yellowish oil (β/α ratio 3:7, 58%); Rf 0.44 (EtOAc/MeOH 9/1); ^1^H-NMR (CDCl_3_): 5.07–4.78(m, 2H), 4.03–3.89 (m, 4H), 3.65–3.60 (m, 2H), 3.47–3.39 (m, 2H), 2.39–2.34 (m, 4H), 1.63–1.58 (m, 4H), 1.31–1.23 (m, 30H), 0.94 (m, 6H, CH_3_) ^13^C-NMR (CDCl_3_): δ 174.5, 172.1, 93.7, 93.0, 75.9, 74.1, 73.6, 72.6, 71.2, 69.8, 68.6, 68.0, 34.4, 34,3, 32.0–21.6 (CH_2_ alkyl chain), 17.7, 17.5, 13.4. Elemental Analysis; calculated for C_16_H_30_O_6_: C, 60.35; H, 9.50; O, 30.15; found: C, 59.23; H, 9.30; O, 31.47.

#### 4.3.4. Octenylsuccinate α/β-_L_-Rhamnopyranoside (Rh-Suc-C8)

Colorless oil (β/α ratio 2:8, 59%); Rf 0.19 (EtOAc/MeOH 9/1); ^1^H-NMR (CDCl_3_): 5.47 (m, 2H), 5.29 (m, 2H), 5.20–5.14 (m, 2H), 4.07–4.03 (m, 2H), 3.69–3.64 (m, 4H), 3.44–3.41 (m, 4H), 2.89–2.84 (m, 2H), 2.71–2.62 (m, 4H), 2.23–1.98 (m, 8H), 1.32–1.08 (m, 18H), 0.91 (m, 6H, CH_3_) ^13^C-NMR (CDCl_3_): δ 178.2, 175.6, 174.8, 134.8, 125.0, 94.4, 91.0, 74.2, 73,8, 73.5, 71.0, 70.7, 44.3, 31.7–21.9 (CH_2_ alkyl chain), 16.8, 16.2, 13.8. Elemental Analysis; calculated for C_18_H_30_O_8_: C, 57.74; H, 8.08; O, 34.18; found: C, 57.03; H, 8.28; O, 34.69.

#### 4.3.5. Iso-Dodecenylsuccinate α/β-_L_-Rhamnopyranoside (Rh-Suc-C12b)

Yellowish solid (β/α ratio 2:8, 55%); Rf 0.23 (EtOAc/MeOH 9/1); ^1^H-NMR (CDCl_3_): 5.49 (m, 2H), 5.33 (m, 2H), 5.18–5.11 (m, 2H), 4.09–4.02 (m, 2H), 3.71–3.65 (m, 4H), 3.45–3.41 (m, 4H), 2.90–2.84 (m, 2H), 2.71–2.62 (m, 4H), 2.23–1.98 (m, 8H), 1.65–1.61 (m, 2H), 1.32–1.08 (m, 26H), 0.91 (m, 6H, CH_3_) ^13^C-NMR (CDCl_3_): δ 178.2, 175.6, 174.9, 134.6, 125.2, 94.4, 91.1, 74.1, 73,8, 73.4, 71.0, 70.7, 44.4, 31.8–21.8 (alkyl chain), 17.0, 16.9, 16.7, 16.4, 13.7. Elemental Analysis; calculated for C_22_H_38_O_8_: C, 61.37; H, 8.90; O, 29.73; found: C, 60.97; H, 8.85; O, 30.18.

#### 4.3.6. 3-Octenyloctylsuccinate α/β-_L_-Rhamnopyranoside (Rh-Suc-C8-C8)

Yellowish oil (β/α ratio 2:8, 39%); Rf 0.46 (EtOAc/MeOH 9/1); ^1^H-NMR (CDCl_3_): 5.46 (m, 2H), 5.28 (m, 2H), 5.21–5.12 (m, 2H), 4.09–4.02 (m, 2H), 3.71–3.62 (m, 4H), 3.45–3.40 (m, 4H), 2.90–2.83 (m, 2H), 2.73–2.63 (m, 4H), 2.40–1.98 (m, 12H), 1.61–1.58 (m, 4H), 1.32–1.08 (m, 40H), 0.92 (m, 6H, CH_3_), 0.89 (m, 6H, CH_3_) ^13^C-NMR (CDCl_3_): δ 178.2, 175.6, 174.8, 134.8, 125.0, 94.4, 91.0, 74.2, 73,8, 73.5, 71.0, 70.7, 44.3, 34.4, 34,2, 31.7–21.4 (CH_2_ alkyl chain), 17.4, 17.2, 16.8, 16.2, 13.8, 13.3. Elemental Analysis; calculated for C_26_H_46_O_8_: C, 64.17; H, 9.53; O, 26.30; found: C, 63.46; H, 9.62; O, 26.92.

#### 4.3.7. 3-Octenyldodecylsuccinate α/β-_L_-Rhamnopyranoside (Rh-Suc-C8-C12)

Yellowish oil (β/α ratio 2:8, 38%); Rf 0.45 (EtOAc/MeOH 9/1); ^1^H-NMR (CDCl_3_): 5.46 (m, 2H), 5.28 (m, 2H), 5.21–5.12 (m, 2H), 4.09–4.02 (m, 2H), 3.71–3.62 (m, 4H), 3.45–3.40 (m, 4H), 2.90–2.83 (m, 2H), 2.72–2.63 (m, 4H), 2.41–1.97 (m, 12H), 1.65–1.57 (m, 4H), 1.32–1.08 (m, 56H), 0.92 (m, 12H, CH_3_), ^13^C-NMR (CDCl_3_): δ 178.2, 175.6, 174.8, 134.8, 125.0, 94.4, 91.0, 74.2, 73,8, 73.5, 71.0, 70.7, 44.3, 34.4, 34,3, 32.0–21.6 (CH_2_ alkyl chain), 17.6, 17.3, 16.8, 16.2, 13.8, 13.3. Elemental Analysis; calculated for C_30_H_54_O_8_: C, 66.39; H, 10.03; O, 23.58; found: C, 65.22; H, 9.88; O, 24.90.

#### 4.3.8. 3-Dodecenyloctylsuccinate α/β-_L_-Rhamnopyranoside (Rh-Suc-C12-C8)

Yellowish oil (β/α ratio 2:8, 37%); Rf 0.47 (EtOAc/MeOH 9/1); ^1^H-NMR (CDCl_3_): 5.50 (m, 2H), 5.33 (m, 2H), 5.17–5.10 (m, 2H), 4.09–4.02 (m, 2H), 3.71–3.64 (m, 4H), 3.45–3.41 (m, 4H), 2.92–2.85 (m, 2H), 2.69–2.60 (m, 4H), 2.41–2.34 (m, 4H), 2.23–1.99 (m, 8H), 1.63–1.59 (m, 4H), 1.32–1.06 (m, 56H), 0.92 (m, 12H, CH_3_) ^13^C-NMR (CDCl_3_): δ 178.0, 175.4, 174.6, 134.6, 125.0, 94.4, 91.1, 74.2, 73,9, 73.4, 71.3, 70.9, 44.3, 32.1–21.6 (CH_2_ alkyl chain), 17.7, 17.3, 17.0, 16.6, 13.8, 13.4. Elemental Analysis; calculated for C_30_H_54_O_8_: C, 66.39; H, 10.03; O, 23.58; found: C, 65.58; H, 9.80; O, 24.62.

#### 4.3.9. 3-Dodecenyldodecylsuccinate α/β-_L_-Rhamnopyranoside (Rh-Suc-C12-C12)

Yellowish oil (β/α ratio 2:8, 34%); Rf 0.43 (EtOAc/MeOH 9/1); ^1^H-NMR (CDCl_3_): 5.51 (m, 2H), 5.34 (m, 2H), 5.18–5.09 (m, 2H), 4.09–4.03 (m, 2H), 3.70–3.58 (m, 6H), 3.49–3.41 (m, 6H), 2.91–2.84 (m, 2H), 2.71–2.63 (m, 4H), 2.40–2.32 (m, 4H), 2.25–2.00 (m, 8H), 1.66–1.59 (m, 4H), 1.33–1.08 (m, 72H), 0.92 (m, 12H, CH_3_) ^13^C-NMR (CDCl_3_): δ 178.1, 175.5, 174.8, 134.7, 125.1, 94.5, 91.0, 74.2, 73,9, 73.4, 71.1, 70.8, 44.3, 34.5, 34.2, 31.9–21.5 (CH_2_ alkyl chain), 17.6, 17.3, 16.9, 16.4, 13.9, 13.7. Elemental Analysis; calculated for C_34_H_62_O_8_: C, 68.19; H, 10.44; O, 21.37; found: C, 67.69; H, 9.90; O, 22.41.

### 4.4. Plant Treatment and Inoculation

All in *planta* assays were carried out using the pathogenic *Z. tritici* strain T02596 isolated in 2014 from Northern France, and the susceptible wheat cultivar Alixan (Limagrain, France) showing compatible interaction with the strain T02596 [36]. The experiments were performed in the greenhouse at 21 ± 2 °C with a 16/8 h day/night cycle. Wheat seeds were pre-germinated in square Petri dishes on moist filter paper, as described by Siah et al. [37]. Germinated grains were placed into 3-L pots filled with universal loam (Gamm Vert, France) and put into the greenhouse. For each condition, three pots containing 12 plants each, meaning 36 plants in total, were used as replicates. Three weeks after sowing, when third-leaves are fully expanded, 30 mL of a solution of 1.5 mM of each RL, as well as lauric acid and dodecanol, were hand-sprayed on the plants of each pot. These molecules were first dissolved in dimethyl sulfoxide (DMSO), with final concentration of DMSO at 0.2%, before being supplemented with 0.05% polyoxyethylene-sorbitan monolaurate (Tween 20, Sigma-Aldrich, Saint-Quentin-Fallavier, France) as a wetting agent. Control plants were treated with a solution of 0.2% DMSO, supplemented with 0.05% Tween 20. Due to solubilization difficulties at 1.5 mM, treatments with Rh-Eth-C18 and Rh-Suc-C12-C12 were not performed. Two days after treatment, 30 mL of fungal spore suspension (10^6^ spores·mL^−1^), also supplemented with 0.05% of Tween 20, were hand-sprayed on the plants of each pot. *Zymoseptoria tritici* spores were harvested after being cultivated on potato dextrose agar medium (PDA) plates for one week in dark conditions, according to Siah et al. [37]. Immediately after inoculation, each pot was covered with a clear polyethylene bag for 3 days in order to ensure a high humidity level allowing a good plant infection. Three weeks after inoculation, the disease severity was measured on the third leaf by assessing the area covered by lesions (chlorosis or necrosis) bearing or not pycnidia. This experiment was repeated twice. In the case of Rh-Est-C12, the most efficient RL in our conditions, the protection efficacy of this compound was assessed at different concentrations, ranging from 50 µM to 3200 µM (50, 100, 200, 400, 800, 1600, and 3200 µM). This experiment underwent the same conditions as described above, except for DMSO that was used at 0.1% final concentration in the treatment solutions.

### 4.5. Plant Defence-Related Enzyme Assays

Evaluation of the indirect effect of RLs, as well as lauric acid and dodecanol, was performed by assessing the activity of two antioxidant key-enzymes involved in plant defense mechanisms: catalase (CAT; EC: 1.11.1.6) and peroxidase (POX; EC: 1.11.1.7). Wheat plants grown under greenhouse conditions treated or not with 1.5 mM of RLs, as described above, were used to examine the elicitation activity of the compounds. Plant third-leaves were harvested two days after treatment and 100 mg were immediately frozen in liquid nitrogen and stored at −80 °C for further analyses. Three leaves, sampled randomly from three different pots (one leaf per pot), were used as repetitions for each condition. Leaf segments were later ground with a mortar and pestle in liquid nitrogen. The powder obtained was resuspended in 1 mL of 50 mM ice-cold potassium phosphate buffer, pH 7.0, and centrifuged at 12,000× *g* for 10 min at 4 °C. The supernatant was used to evaluate the activity of CAT or POX. POX activity was assessed based on the procedure of Mitchell et al. [38] and Shindler et al. [39]. The reactive medium consisted of 0.5 mM 2,2′-azino-bis(3-ethylbenzothiazoline-6-sulfonic acid (ABTS) and 0.25 mM H_2_O_2_ in 25 mM sodium acetate buffer, pH 4.4. This assay was performed in flat-bottomed polystyrene 96-well microplates (Corning Costar^®^, Corning, NY, USA). To initiate the reaction, 10 µL of supernatant was added to 190 µL of the reactive mix in plate wells. The increased absorbance at 412 nm, produced by the formation of the radical cation, was monitored kinetically for 5 min at 25 °C. POX activity was expressed as change in absorbance at 412 nm·min^−1^·g^−1^ protein. CAT activity was determined according to Beers and Sizer [40] and García-Limones et al. [41] with slight modifications. To initiate the reaction, 10 µL of wheat leaf supernatant diluted in sterile water 1:4 (*v*/*v*) were added to the wells containing 190 µL of reactive medium consisting of 50 mM H_2_O_2_ in 50 mM potassium phosphate buffer, pH 7.0. 96-well microplates (Greiner UV-Star^®^, Greiner Bio-One GmbH, Kremsmünster, Austria), allowing measurements up to 200 nm, were used. The decreased absorbance at 240 nm resulting from the degradation of H_2_O_2_ was measured kinetically during 5 min at 25 °C. CAT activity was expressed as change in absorbance at 240 nm·min^−1^·g^−1^ protein. Protein content of wheat leaf supernatants was assessed using the method of Bradford [42] with bovin serum albumin (BSA) as a standard.

### 4.6. In Planta Antifungal Effect of Rh-Est-C12

Wheat plants grown under greenhouse conditions, sprayed or not with different concentrations of Rh-Est-C12 (0, 50, 400, and 3200 µM) and inoculated with spore suspension of the T02596 Z. tritici strain, were used to assess *in planta* the activity of this molecule on spore germination and hyphal growth of the phytopathogen. The three concentrations 50, 400, and 3200 µM were chosen because they correspond to the lowest, median, and highest concentrations tested *in planta* for this RL, respectively. The chitin staining dye Fluorescent Brightener 28 (Calcofluor, Sigma-Aldrich, USA) was used to stain the fungus on the leaf surfaces, according to Siah et al. [37]. Three days after inoculation, wheat third-leaf segments (2 cm) were harvested and immersed for five minutes in a solution of 0.1% (*w*/*v*) Calcofluor and 0.1 M Tris-HCl buffer, pH 8.5. Leaf segments were then washed twice in sterile osmosed water for two minutes. After being dried in dark conditions at room temperature, they were placed on a glass slide, covered with a cover slip and observed microscopically (Eclipse 80i, Nikon, Champigny-sur-Marne, France) under UV-light. For each leaf segment, the percentage of different spore classes was calculated on 100 observed spores (class 1: non germinated spore; class 2: geminated spore with a small germ tube; class 3: geminated spore with a developed germ tube; class 4: geminated spore with a strongly developed germ tube). Six third-leaf segments from three different pots (two segments per pot) were used as replicates for each condition. Pictures were taken with a digital camera (DXM1200C, Nikon, Champigny-sur-Marne, France) using image capture software (NIS-Elements BR, Nikon, Champigny-sur-Marne, France).

### 4.7. In Vitro Direct Antifungal Effect of Rhamnolipids

In vitro antifungal activity of all tested RLs, as well as dodecanol and lauric acid, was assessed by measuring the growth of *Z. tritici* colonies on solid medium in sterile 12-well plates (Cellstar standard^®^, Greiner Bio-One GmbH, Kremsmünster, Austria). Plate wells were filled with 3 mL of PDA, autoclaved beforehand, mixed with our molecules of interest at approximatively 40 °C. The compounds were first dissolved in DMSO (0.1% final concentration in the wells). After PDA cooling down, 5 µL of the *Z. tritici* strain T02596 spore suspension (5 × 10^5^ spores·mL^−1^) were added to each well, according to Siah et al. [37]. Ten different concentrations (39.5, 59.3, 88.9, 133.3, 200, 300, 450, 666.7, 1000, and 1500 mM) were used for each molecule. Moreover, control mediums supplemented with 0.1% of DMSO, with or without fungal inoculation, were used. After inoculation, plates were incubated for 10 days in growth chamber in dark conditions at 20 ± 1 °C, after which two perpendicular diameters of fungal colonies were measured visually. This experiment was repeated three times and three wells were used as replicates for each condition.

### 4.8. Fungal Strain Effect of Rh-Est-C12 Antifungal Activity

To assess the fungal strain effect of the most active RL Rh-Est-C12 regarding its antifungal direct activity, a total of 21 single-conidial *Z. tritici* strains (Zt.1 to Zt.21) isolated in 2016 from the Arras location in northern France, and the reference strain IPO323 isolated in The Netherlands in 1981 [43], were assessed for their variability in sensitivity to Rh-Est-C12. These *Z. tritici* strains were chosen because they displayed, in preliminary assays, different resistance levels towards five fungicide molecules belonging to DMI class (tebuconazole, metconazole, epoxiconazole, prothioconazole, prochloraz). The sensitivity of the strains towards these fungicides was evaluated in 96-well microplates, as described in Siah et al. [37], at the concentrations 75, 25, 8.33, 2.78, 0.93, 0.31, 0.10, and 0.03 mg·L^−1^. The antifungal bioassay for Rh-Est-C12 against the 22 *Z. tritici* strains was performed at the concentrations 450, 300, 200, 133.3, 88.9, 59.3, and 39.5 µM.

### 4.9. Statistical Analyses

Data obtained for *in planta* protection efficacy, enzyme activity, and *in planta* spore germination assays were subjected to analysis of variance (ANOVA) followed by Tukey’s test at *p* ≤ 0.05, using the XLSTAT software (Addinsoft, Paris, France). For in vitro antifungal activity assays, the minimal inhibitory concentration (MIC), defined as the minimal tested concentration totally inhibiting fungal growth, was scored for each molecule. In addition, half-maximal inhibitory concentration (IC_50_) was calculated using GraphPad Prism software version 8 (GraphPad Software Inc., San Diego, CA, USA) for each molecule and tested fungal strain. In the strain effect assay, resistance factors were calculated by dividing the IC_50_ of each strain by the IC_50_ of the reference strain IPO323 for each tested compound. Correlations between protection efficacies, in vitro direct activity (IC_50_ and MIC), and *in planta* indirect effect (CAT and POX activities) within all the tested molecules, was carried out with principal component analysis (PCA) based on the Pearson correlation test, using XLSTAT software (Addinsoft).

## Figures and Tables

**Table 1:** List, structure and characteristics of the eighteen synthesized RLs as well as lauric acid, dodecanol and natural RLs.

Scheme	Compound	Name	Molecular Weight (g moL^−1^)
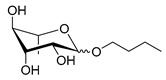	Rh-Eth-C4	Butyl *α*/*β*-_L_-rhamnopyranoside	220
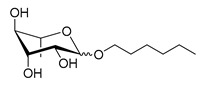	Rh-Eth-C6	Hexyl α/β-_L_-rhamnopyranoside	248
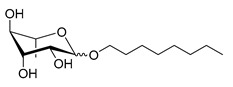	Rh-Eth-C8	Octyl α/β-_L_-rhamnopyranoside	276
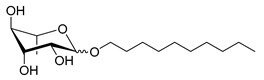	Rh-Eth-C10	Decyl α/β-_L_-rhamnopyranoside	304
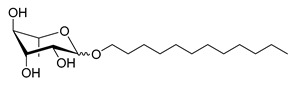	Rh-Eth-C12	Dodecyl α/β-_L_-rhamnopyranoside	332
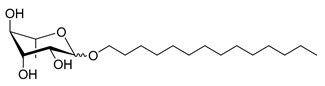	Rh-Eth-C14	Tetradecyl α/β-_L_-rhamnopyranoside	360
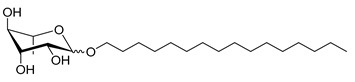	Rh-Eth-C16	Hexadecyl α/β-_L_-rhamnopyranoside	388
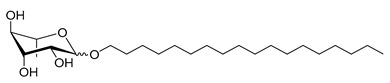	Rh-Eth-C18 *	Octadecyl α/β-_L_-rhamnopyranoside	416
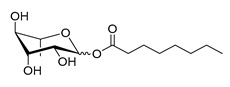	Rh-Est-C8 *	Octanoyl α/β-_L_-rhamnopyranoside	290
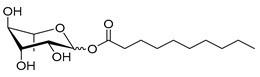	Rh-Est-C10 *	Decanoyl α/β-_L_-rhamnopyranoside	318
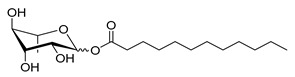	Rh-Est-C12	Dodecanoyl α/β-_L_-rhamnopyranoside	346
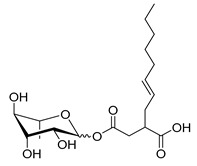	Rh-Suc-C8 *	Octenylsuccinate α/β-_L_-rhamnopyranoside	374
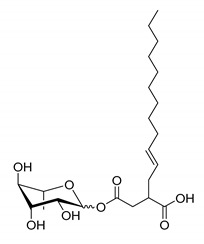	Rh-Suc-C12	Dodecenylsuccinate α/β-_L_-rhamnopyranoside	430
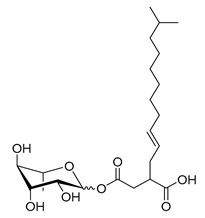	Rh-Suc-C12b *	*Iso*-Dodecenylsuccinate α/β-_L_-rhamnopyranoside	430
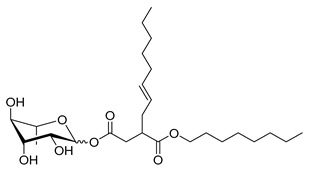	Rh-Suc-C8-C8 *	3-Octenyloctylsuccinate α/β-_L_-rhamnopyranoside	486
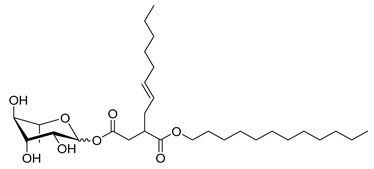	Rh-Suc-C8-C12 *	3-Octenyldodecylsuccinate α/β-_L_-rhamnopyranoside	542
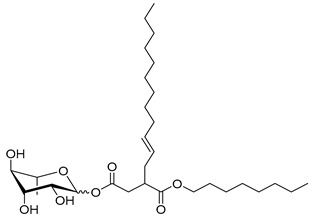	Rh-Suc-C12-C8 *	3-Dodecenyloctylsuccinate α/β-_L_-rhamnopyranoside	542
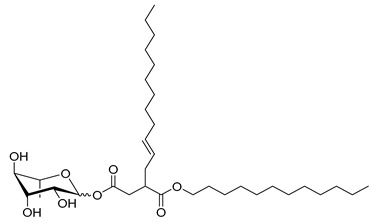	Rh-Suc-C12-C12 *	3-Dodecenyldodecylsuccinate α/β-_L_-rhamnopyranoside	598
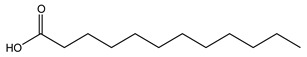	Lauric acid	Lauric acid	200
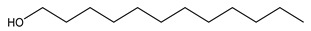	Dodecanol	Dodecanol	186
Mixture of rhamnolipids (data from Agae Technologies LLC)	Nat-Rh	Bacterial rhamnolipids (see Section 4)	578

* Molecules newly synthetized in this study, while the other molecules were already reported in Robineau et al. [25].

**Table 2:** Biological activities, including in vitro direct antifungal activity, *in planta* defense activation and *in planta* protection efficacy of the eighteen synthetized RLs, as well as lauric acid, dodecanol and natural RLs, at 1.5 mM, on the wheat-*Zymoseptoria tritici* pathosystem.

Molecule	In Vitro Antifungal Activity		*In Planta* Defense Elicitation Activity		*In Planta* Protection Efficacy
	IC_50_ (μM)	MIC (μM)	Catalase Activity (U min^−1^ g^−1^ Proteins)	Peroxidase Activity (U min^−1^ g^−1^ Proteins)	Leaf Diseased Area (%) ^•^
Control	-	-	83.4 ^ab^	51.7 ^bc^	55.7 ^a^ (0%)
Natural-Rh	>1500	>1500	71.9 ^a^	53.4 ^c^	**46.3 ^b^ (−17.0%)**
Rh-Eth-C4	>1500	>1500	110.4 ^abcd^	64.8 ^bc^	51.4 ^ab^ (−7.8%)
Rh-Eth-C6	>1500	>1500	111.7 ^abcd^	55.9 ^bc^	54.4 ^ab^ (−2.3%)
Rh-Eth-C8	>1500	>1500	121.1 ^abcd^	74.2 ^abc^	54.3 ^ab^ (−2.6%)
Rh-Eth-C10	**158**	**200**	131.6 ^bcd^	54.2 ^bc^	**46.0 ^b^ (−17.4%)**
Rh-Eth-C12	**81**	**450**	**155.2 ^d^**	71.0 ^abc^	**46.2 ^b^ (−15.5%)**
Rh-Eth-C14	>1500	>1500	99.2 ^abcd^	71.2 ^abc^	49.4 ^ab^ (−11.3%)
Rh-Eth-C16	>1500	>1500	130.9 ^bcd^	67.7 ^bc^	50.3 ^ab^ (−9.7%)
Rh-Eth-C18	>1500	>1500	Nd *	Nd *	Nd *
Rh-Est-C8	>1500	>1500	112.9 ^abcd^	44.9 ^c^	50.6 ^ab^ (−9.1%)
Rh-Est-C10	>1500	>1500	98.4 ^abcd^	58.2 ^bc^	50.6 ^ab^ (−9.3%)
Rh-Est-C12	**75**	**133**	**142.5 ^cd^**	**117.3 ^a^**	**35.9 ^c^ (−35.6%)**
Rh-Suc-C8	**1399**	**1500**	123.3 ^abcd^	60.3^bc^	54.1 ^ab^ (−3.0%)
Rh-Suc-C12	>1500	>1500	124.9 ^abcd^	76.6 ^abc^	50.3 ^ab^ (−9.7%)
Rh-Suc-C12b	>1500	>1500	96.8 ^abc^	**116.9 ^a^**	**46.2 ^b^ (−16.2%)**
Rh-Suc-C8-C8	>1500	>1500	102.4 ^abcd^	62.1 ^bc^	52.4 ^ab^ (−6.0%)
Rh-Suc-C8-C12	>1500	>1500	113.7 ^abcd^	96.9 ^ab^	51.2 ^ab^ (−8.1%)
Rh-Suc-C12-C8	>1500	>1500	120.1 ^abcd^	79.9 ^abc^	55.8 ^a^ (+0.1%)
Rh-Suc-C12-C12	>1500	>1500	Nd *	Nd *	Nd *
Lauric acid	**109**	**200**	94.0 ^abc^	76.8 ^abc^	50.9 ^ab^ (−8.7%)
Dodecanol	**164**	**200**	107.4 ^abcd^	51.1 ^bc^	54.4 ^ab^ (−2.4%)

(*) No data were obtained for two RLs because of solubilization issues. Different letters indicate significant differences between treatments according to ANOVA followed by Tukey’s test at *p* ≤ 0.05. Values in bold indicate significant effects. (^•^) Values between brackets stand for the percentage of disease symptom reduction compared to the control condition.

**Table 3:** Correlations between protection efficacy and both direct (IC_50_ and MIC) and indirect (plant catalase and peroxidase activities) effects of the tested RLs, as well as dodecanol and lauric acid, obtained on the wheat-*Zymoseptoria tritici* pathosystem.

	IC_50_	MIC	Catalase Activity	Peroxidase Activity
IC_50_	-	-	-	-
MIC	**0.99**	-	-	-
Catalase activity	−0.40	−0.35	-	-
Peroxidase activity	−0.10	−0.10	0.19	-
Leaf diseased area	**0.46**	**0.46**	−0.25	**−0.51**

Significant values according to the Pearson test at *p* ≤ 0.05 are indicated in bold.

## Data Availability

The data presented in this study are available in article and supplementary material.

## Supplementary Materials

The Supplementary Materials are available online. Supplementary Table S1: Half-maximal inhibitory concentration (IC_50_) values obtained for 22 *Zymoseptoria tritici* strains differing in their resistance level to DMI fungicides, towards five DMI molecules and the rhamnolipid molecule Rh-Est-C12. Supplementary Table S2: Resistance factors obtained for 22 *Zymoseptoria tritici* strains differing in their resistance level to DMI fungicides, towards five DMI molecules and the rhamnolipid molecule Rh-Est-C12, calculated from the IC_50_ values presented in the Supplementary Table 1. Resistance factors correspond to the ratio between the IC_50_ value of a given strain and the IC_50_ value of the sensitive reference strain IPO323.

## Sample Availability

Samples of the compounds are not available from the authors.
