# Supplementary material for: Importance of the C12 Carbon Chain in the Biological Activity of Rhamnolipids Conferring Protection in Wheat against Zymoseptoria tritici
Source: Molecules. 2020 Dec 23;26(1):40. doi: 10.3390/molecules26010040 (PMC7796335; doi:10.3390/molecules26010040)
Supplement: Supplementary file 1 [file molecules-26-00040-s001.pdf]

molecules MDPI

Supplementary Table S1. Half-maximal inhibitory concentration (IC_{50}) values obtained for 22 *Zymoseptoria tritici* strains differing in their resistance level to DMI fungicides, towards five DMI molecules and the rhamnolipid molecule Rh-Est-C12.

***Zymoseptoria tritici* isolates**		IC_{50} **(mg.L**$^{-1}$**)**					
		Tebuconazole	Metconazole	Epoxiconazole	Prothioconazole	Prochloraz	Rh-Est-C12
**Control strain**	IPO323	0.07	0.04	0.03	0.001	0.02	**54.3**
**No MDR or *Cyp51* gene overexpressing strains**	Zt.1	0.26	0.14	1.12	0.10	0.12	**49.9**
	Zt.2	0.54	0.13	1.39	0.10	0.17	**65.4**
	Zt.3	0.49	0.20	1.22	0.09	0.16	**64.1**
	Zt.4	0.48	0.25	1.18	0.14	0.17	**50.3**
	Zt.5	0.38	0.25	1.63	0.12	0.12	**59.0**
	Zt.6	0.36	0.18	1.35	0.08	0.17	**47.4**
	Zt.7	0.29	0.14	1.03	0.12	0.20	**60.3**
***Cyp51* gene overexpressing strains**	Zt.8	5.42	0.25	1.05	0.12	0.19	**49.7**
	Zt.9	9.42	0.61	1.45	0.11	0.12	**49.3**
	Zt.10	15.54	0.56	1.69	0.05	0.39	**44.2**
	Zt.11	12.02	0.72	1.62	0.05	0.20	**51.7**
	Zt.12	18.85	1.10	1.48	0.08	0.25	**48.7**
	Zt.13	21.07	1.35	3.63	0.05	0.79	**49.2**
	Zt.14	2.33	0.27	0.86	0.04	0.20	**45.3**
**MDR strains**	Zt.15	5.91	1.30	3.82	0.28	3.66	**49.2**
	Zt.16	4.86	1.27	7.47	75.00	3.13	**44.2**
	Zt.17	5.26	0.04	6.72	0.28	1.94	**40.0**
	Zt.18	3.53	1.05	8.03	0.30	1.28	**32.0**
	Zt.19	75.00	6.02	15.05	0.57	1.48	**56.6**
	Zt.20	15.51	3.17	10.56	0.04	6.38	**47.5**
	Zt.21	4.82	1.54	7.61	0.33	2.86	**46.2**

Supplementary Table S2. Resistance factors obtained for 22 *Zymoseptoria tritici* strains differing in their resistance level to DMI fungicides, towards five DMI molecules and the rhamnolipid molecule Rh-Est-C12, calculated from the IC_{50} values presented in the Supplementary Table S1.

***Zymoseptoria tritici* isolates**		**Resistance factors ***					
		Tebuconazole	Metconazole	Epoxiconazole	Prothioconazole	Prochloraz	Rh-Est-C12
**Control strain**	IPO323	1 (0.07)	1 (0.04)	1 (0.03)	1 (0.001)	1 (0.02)	**1 (54.29)**
**No MDR or *Cyp51* gene overexpressing strains**	Zt.1	3.8	3.6	37.2	102.4	5.1	**0.9**
	Zt.2	7.6	3.3	46.2	104.4	7.2	**1.2**
	Zt.3	7.0	4.9	40.7	89.3	6.7	**1.2**
	Zt.4	6.8	6.2	39.5	137.5	7.2	**0.9**
	Zt.5	5.4	6.1	54.3	117.0	5.1	**1.1**
	Zt.6	5.2	4.6	45.2	84.5	6.9	**0.9**
	Zt.7	4.1	3.5	34.2	117.8	8.2	**1.1**
***Cyp51* gene overexpressing strains**	Zt.8	77.6	6.3	35.0	123.3	8.1	**0.9**
	Zt.9	134.6	15.3	48.2	112.8	5.2	**0.9**
	Zt.10	222.0	14.0	56.3	54.3	16.5	**0.8**
	Zt.11	171.7	18.0	53.9	46.9	8.3	**1.0**
	Zt.12	269.3	27.6	49.3	75.9	10.6	**0.9**
	Zt.13	301.0	33.8	121.1	53.8	33.0	**0.9**
	Zt.14	33.3	6.8	28.6	42.5	8.5	**0.8**
**MDR strains**	Zt.15	84.4	32.5	127.3	276.5	152.6	**0.9**
	Zt.16	69.5	31.7	249.1	74753.2	130.6	**0.8**
	Zt.17	75.1	1.0	224.1	274.3	80.8	**0.7**
	Zt.18	50.4	26.3	267.5	298.6	53.5	**0.6**
	Zt.19	1071.4	150.8	501.8	564.9	61.8	**1.0**
	Zt.20	221.5	79.3	351.9	37.4	266.2	**0.9**
	Zt.21	68.9	38.6	253.5	328.6	119.2	**0.9**

* Resistance factors correspond to the ratio between the IC50 value of a given strain and the IC50 value of the sensitive reference strain IPO323.
